# Synthesis, Characterization, Corrosion Resistance and In-Vitro Bioactivity Behavior of Biodegradable Mg–Zn–Mn–(Si–HA) Composite for Orthopaedic Applications

**DOI:** 10.3390/ma11091602

**Published:** 2018-09-03

**Authors:** Chander Prakash, Sunpreet Singh, Munish Kumar Gupta, Mozammel Mia, Grzegorz Królczyk, Navneet Khanna

**Affiliations:** 1 School of Mechanical Engineering, Lovely Professional University, Phagwara, Punjab 144411, India; chander.mechengg@gmail.com (C.P.); snprt.singh@gmail.com (S.S.); 2 Mechanical Engineering Department, National Institute of Technology, Hamirpur 177005, India; munishguptanit@gmail.com; 3 Mechanical and Production Engineering, Ahsanullah University of Science and Technology, Dhaka 1208, Bangladesh; 4 Department of Manufacturing Engineering and Automation, Opole University of Technology, 76 Proszkowska St., 45-758 Opole, Poland; g.krolczyk@po.opole.pl; 5 Mechanical Engineering, Institute of Infrastructure, Technology, Research and Management (IITRAM), Gujarat 380026, India; navneetkhanna@iitram.ac.in

**Keywords:** magnesium, alloying, spark plasma sintering, elastic modulus, corrosion resistance, bioactivity

## Abstract

Recently, magnesium (Mg) has gained attention as a potential material for orthopedics devices, owing to the combination of its biodegradability and similar mechanical characteristics to those of bones. However, the rapid decay rate of Mg alloy is one of the critical barriers amongst its widespread applications that have provided numerous research scopes to the scientists. In this present, porous Mg-based biodegradable structures have been fabricated through the hybridization of elemental alloying and spark plasma sintering technology. As key alloying elements, the suitable proportions of silicon (Si) and hydroxyapatite (HA) are used to enhance the mechanical, chemical, and geometrical features. It has been found that the addition of HA and Si element results in higher degree of structural porosity with low elastic modulus and hardness of the Mg–Zn–Mn matrix, respectively. Further, addition of both HA and Si elements has refined the grain structure and improved the hardness of the as-fabricated structures. Moreover, the characterization results validate the formation of various biocompatible phases, which enhances the corrosion performance and biomechanical integrity. Moreover, the fabricated composites show an excellent bioactivity and offer a channel/interface to MG-63 cells for attachment, proliferation and differentiation. The overall results of the present study advocate the usefulness of developed structures for orthopedics applications.

## 1. Introduction

The demand of artificial organs and other biomedical devices has increased drastically during the recent decades. Commonly used and successful implant materials are stainless steel (SS), cobalt–chromium (Co–Cr), titanium (Ti) and their alloys/composites [1]. However, they may have exceeded their full potential because of their drawbacks. Firstly, the Young’s modulus of the aforementioned materials (110–200 GPa) is higher than that of the bone (7–25 GPa), which causes stress shielding [2]. As a result, the bone resorption occurs, which causes implant loosening and failure. Secondly, the implants made up of these biomaterials are unbiodegradable and after bone healing, the implants should be taken out from the body by performing a second surgery [3]. Due to adverse repercussions of non-degradable materials, feasibility of developing biodegradable materials has attracted the greatest interest. Recently, magnesium (Mg) and its alloys have gained a great deal of attention as a promising and potential biodegradable material for the fabrication of bone fixation accessories, because of their high biological properties [4]. However, the poor corrosion resistance of Mg is one of the most critical barriers, owing to which it degrades very rapidly after implantation [5,6]. Over the years, several methodologies were used to control the degradation rate of Mg and its alloys [7,8,9]. Elemental alloying has been reported as the most effective technique to improve corrosion resistance and mechanical properties of Mg alloys [10]. 

Elemental alloying is a powder metallurgical process, in which metallic powder particles are mechanically alloyed and subsequently sintered by appropriate techniques. In the past, alloying of safe elements, such as zinc (Zn), aluminium (Al), silver (Ag), yttrium (Y), zirconium (Zr), neodymium (Nd), silicon (Si), manganese (Mn), titanium dioxide (TiO_2_), and calcium (Ca), were selected in response to increase the corrosion resistance and biological function of Mg [11,12,13,14,15,16,17]. Although the element Al in Mg composites improves mechanical properties, released Al^+^ ions cause Alzheimer’s disease and muscle fiber damage [18,19,20]. It was reported that Zr causes very serious diseases, such as liver, lung, and breast cancer [21]. Zhang et al. [22] reported that alloying Nd and Y in Mg alloys disrupt the growth of tissues around the implant. Li et al. [23] observed that the alloying of Ca reduces the degradation rate and improves biomechanical integrity in a corrosive medium. Moreover, Ca is a base element of human bone, which stimulates the new tissue growth and accelerates the bone healing process. The alloying of Zn and Mn in the Mg matrix enhances both elasticity and corrosion resistance [10]. Recently, Ben-Hamu et al. [24] reported that Si has proved to be an essential element being alloyed to develop tissues and immune systems. The developed Mg–Si composites possess required mechanical properties, low ductility, and high strength. Moreover, polygonal-shape Mg_2_Si intermetallics inhibit the corrosion more effectively compared to the Chinese script.

Recently, the application of spark plasma sintering (SPS) technique for the synthesis of Mg-based alloys and composites with improved mechano-biological, antibacterial and corrosion performance has been reported. Sunil et al. [25] developed biodegradable Mg–hydroxyapatute (HA) composites by the SPS technique and studied the consequence of HA weight % on corrosion resistance of the developed composites. The Mg–10%HA composite exhibits best corrosion resistance and high hardness. Zheng et al. [26] synthesized a Mg–Al–Zn alloy by SPS, which possesses a maximum microhardness of 140 HV, a compressive yield strength of 442.3 MPa, and an ultimate strength of 546 MPa, which are comparatively higher than those values of conventional Mg alloys. Zhang et al. [27] studied the effect of Ca and Zn on a Mg–Si composite, and it was found that the addition of Ca and Zn to the Mg–Si alloy improved the bio-corrosion resistance and shows very good biocompatibility. In vitro analysis revealed that excellent adhesion and growth of osteoblastic cell has been observed and in vivo results suggested that the alloy has good biocompatibility. The Mg–Zn–Mn–Ca alloy developed by the elemental alloying and SPS technique exhibits high yield strength (58–69 MPa), strong tensile strength (177–205 MPa), and strong hardness (49–53 Hv) [28]. The effect of HA along with Zn and Mn on the microstructure, corrosion performance and mechanical properties of Mg alloy was reported. The alloying of HA (5 wt %) improves the corrosion resistance of Mg [29,30]. Further, Prakash et al. [31] investigated the effect of mechanical alloying-assisted SPS process (MA-SPS) parameters on structural porosity, elastic modulus, and hardness of the composite. Multi-objective particle swarm optimization (MO-PSO) has been utilized to determine the optimal setting of MA-SPS to sinter mechanically tuned biocompatible composites with improved corrosion properties.

It is clear that many studies, in the past, reported on design, development and synthesis of Mg alloy alloyed with Mn, and Zn using various fabrication techniques, with the aim of controlling the degradation rate. However, to the best of authors’ knowledge, limited work is available on hybrid alloying of Si and HA and their effects on mechanical, corrosion properties, degradation and bioactivity analysis of Mg–Zn–Mn alloys. This paper is aimed at studying the synthesis, characterization, corrosion and cell response of Mg–Zn–Mn–(Si–HA) composites fabricated via the MA-SPS technique. The key expectation is that the fabricated porous composite will exhibit an improved biomechanical integrity while offering increased corrosion resistance to delay the degradation and improved bioactivity for orthopedic applications.

## 2. Materials and Methods 

### 2.1. Mechanical Alloying and Consolidation of Spark Plasma Sintering

**Figure 1:**
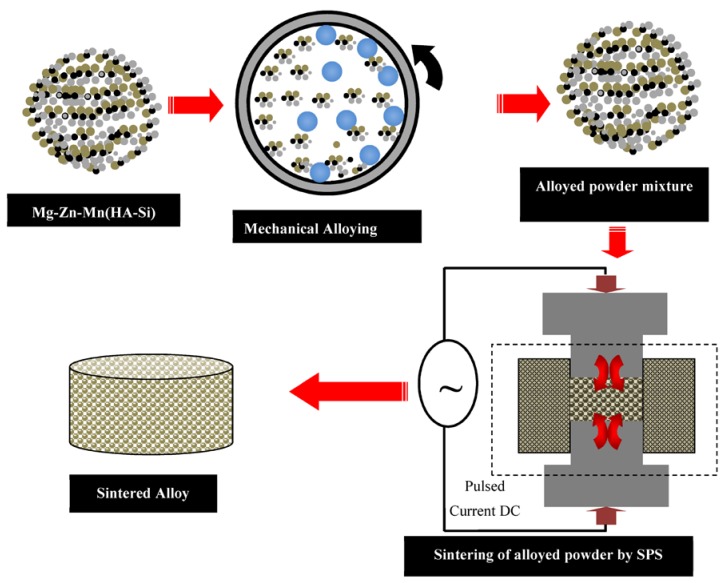
Fabrication route for synthesis of the Mg–Zn–Mn–(HA–Si) alloy.

In this work, high-purity (~99.9%) elemental powders of Mg, Mn, Zn, Si, and HA were used to synthesize Mg–Zn–Mn–Si–HA composites. The chemical composition of the proposed bio-composites in wt % is listed in Table 1. The required powders were weighed and MA has been carried out using planetary ball mill (Fritsch Pulverisette 7, M/s. Fritsch, Germany.) with SS vial and SS balls with a diameter of 5 mm. The powder mixture was mechanically alloyed for about 12 h at 300 rpm with a ball/powder ratio of 10/1. Stearic acid (0.1 gm) was used to prevent agglomeration and excessive cold welding of powders. The blended powders were preheated at 100 °C for 1 h, in the argon atmosphere, in order to remove the moisture. Then, the blended powder was consolidated by the SPS process (SPS-5000 machine; model: Dr. Sinter SPS-625, Fuji Electronic Industrial Co. Ltd., Tsurugashima, Japan). The SPS was carried out at a heating rate of 50 °C /min (for a holding time of 5 min), under vacuum, and at different sintering temperatures and pressure conditions as illustrated in Table 2, as per the procedure reported elsewhere [29,30,31]. Figure 1 presents the fabrication route for the synthesis of Mg–Zn–Mn–(HA–Si) alloy. A graphite die was used for the sintering and the solid compacts of 20 mm in diameter and 4 mm in thickness were synthesized. The objective of changing the temperature and pressure level is to investigate their effect on the porosity, relative density, elastic modulus, and micro-hardness.

### 2.2. Metallurgical and Mechanical Characteristics

The grain size and the lattice-strain of mechanically alloyed powder were determined by the Williamson–Hall method, as expressed in Equation (1):

(1) $$B\mathrm{Cos}\theta =\frac{K\lambda }{D}2\epsilon \mathrm{Sin}\theta$$

where D is the crystal size, K is the shape factor (assume to be 0.9), λ is the wavelength of X-ray, B is the full width at half maximum, ε is the lattice strain, and θ is the Bragg angle [32,33].

The samples for microstructure examination were cut from the sintered compacts by low-speed diamond cutter, and then samples were well polished using emery paper, diamond paste, and napped cloth. The microstructure and morphology of composite were investigated by FE-SEM (Field-Emission Scanning Electron Microscope). The elemental composition was determined with an EDS (Energy Dispersive Spectrometer) detector coupled with the FE-SEM. The phases present in the synthesized composites were studied by X-ray diffraction (XRD) with CuKα radiation at an incident angle range of 20–80°. The elastic modulus and hardness of the as-developed composites were determined via a nano-indentation technique (model: Hyistron TI-950 indentation system, Bruker’s, Minneapolis, MN, USA) via the Oliver–Pharr approach by using the Berkovich tip at 1000 μN [34].

### 2.3. Potentiodynamic Corrosion and Degradation Test 

The corrosion characteristics of the as-synthesized composites were analysed by the potentiodynamic polarization test through an electrochemical system (Gamry 1000E, Potentiostat/Galvanostat, Gamry Instruments, Warminster, PA, USA) in simulated body fluids (9 g/L NaCl, 0.24 g/L CaCl_2_, 0.43 g/L KCl, and 0.2 g/L NaHCO_3_ at pH 7.2). The as-synthesized specimens, graphite rode, and saturated-carmol-electrode (SCE) were treated as the test electrode, the counter, and the reference electrode, respectively. The tests were performed at 37 °C to simulate the physiological environment. The corrosion characteristics were determined according to the approach reported in previous studies [35,36]. The simulated body fluid (SBF) test was conducted to find out the degradation rate of the specimens after 3, 7, and 14 days. The samples were well polished and dipped into the SBF solution in sterilized vials as per the ASTM-G31-72 standard, as reported in [31,37]. After a predetermined time period of immersion, the samples were retrieved from the glass vial, cleaned by water, and dehydrated into the desiccators for 24 h. The degradation rate was determined by the weight loss due to Mg^2+^ ion release in the SBF solution. The degraded surface was investigated by the FE-SEM and ESD techniques. The degradation behavior of as-synthesized composites was also measured and determined by the released concentration of Mg^2+^ molecules/ions in physiological environment during the immersion test, as per the procedure adopted elsewhere [38].

### 2.4. In Vitro Bioactivity Test 

The cell culture, MTT, and differentiation assays were performed to examine the bioactivity and biocompatibility of the as-sintered porous composites using human MG-63 osteoblasts cell lines. The samples were sliced into 5 mm in diameter and 3 mm in thickness according to the geometry of a 96-well culture plat. The cells were cultured in a flask containing Dulbecco's Modified Eagle Medium supplemented with 10% bovine serum Sigma-Aldrich, (SIGMA, St. Louis, MO, USA) and 1 vol % penicillin (Invitrogen, Thermo Fisher Scientific corporation, Waltham, MA, USA) in an incubator at 37 °C and 5% CO_2_ until confluent. The confluent cells were seeded on the Mg composites at a cell density of 1 × 10^5^ cells/cm^2^. The cell proliferation was evaluated using MTT assay (3-(4,5-dimethylthiazol-2-yl)-2,5-diphenyltetrazolium bromide) based on the conversion of MTT substrate to formazan by viable cells. At given time points, the culture medium was removed, and the MTT reagent (50 mL per well, thiazolyl blue tetrazolium bromide (M2128, Sigma-Aldrich, SIGMA, St. Louis, MO, USA) was added to the culture plate and incubated at 37 °C for 4 h. Then, the MTT reagent was removed and dimethyl sulfoxide (50 mL) was added to each well to dissolve the formazan crystals. The results of the MTT assay were expressed as a measure of optical density that was determined at a wavelength of 570 nm. Cell proliferation was also evaluated by determining the DNA content [39]. For staining the live cells, acetoxymethyl (AM) ester (Calcein, Molecular Probes, Crailsheim, Germany) was used, which is a fluorescent indicator. The cell distribution growth on the sample surface was analyzed using a florescent microscope (FM, Scope. A1, Carl Zeiss, Thornwood, NY, USA). After the cultivation period of 48 h, the adherent cells were fixed with 3.7 vol % paraformaldehyde for 10 min and permeabilized with 0.1 vol % Triton X-100 (in PBS) for 10 min at room temperature [40]. At incubation periods of 1, 3 and 7 days, the cultured-specimens were withdrawn from the physicological environment and subjected to fixation using the glutaraldehyde solution and then dehydrated using a series of ethanol. Cell differentiation was evaluated using cellular alkaline phosphatase-specific activity [orthophosphoric monoester phosphohydrolase, alkaline; E.C. 3.1.3.1] as an early differentiation marker and osteocalcin content in the conditioned media as a late differentiation marker. Alkaline phosphatase activity was assayed from the release of p-nitrophenol from p-nitrophenylphosphate at pH 10.2, as previously described. Activity values were normalized to the protein content, which was detected as colorimetric cuprous cations in biuret reaction (BCA Protein Assay Kit, Pierce Biotechnology Inc., Rockford, IL, USA) at 570 nm (Microplate reader, BioRad Laboratories Inc., Hercules, CA, USA) [41]. All experiments were repeated three times to ensure validity of the observations. Analysis of variance (ANOVA) and the significant difference between groups was determined using the Student’s *t* test at a 95% confidence interval. A *p* value of less than 0.05 was considered as statistically significant.

## 3. Results and Discussion

### 3.1. Powder Morphology

**Figure 2:**
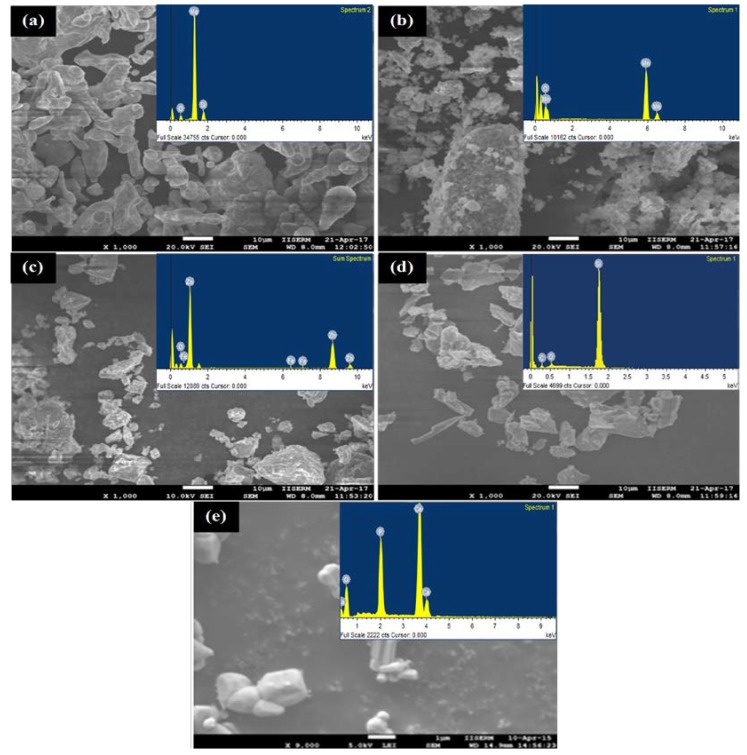
SEM micrographs and EDS spectra of raw powders: (**a**) magnesium; (**b**) manganese; (**c**) zinc; (**d**) silicon and (**e**) hydroxyapatite.

**Figure 3:**
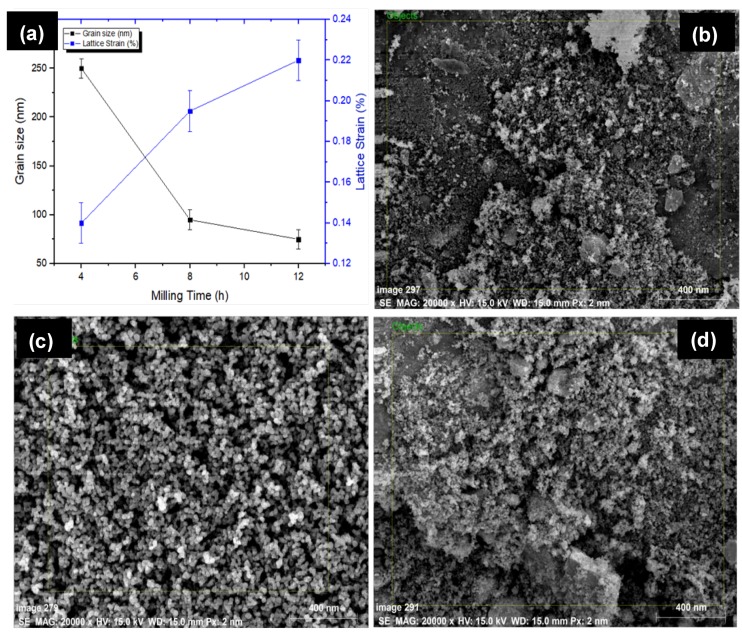
(**a**) Evaluation of grain size and lattice strain as a function of time; powder morphology and size after ball milling for 12 h: (**b**) Mg–Zn–Mn–Si; (**c**) Mg–Zn–Mn–HA; (**d**) Mg–Zn–Mn–Si–HA.

Figure 2 presents the SEM micrograph and associated EDS spectrum of powder particles before MA. The HA powder particles used were of 0.5 μm (irregular), whereas others exhibited spherical morphology with an average size of 25 μm. It has been found that there was no powder loss incurring during the alloying process as the sample size before and after the alloying was recorded to be 10 gm. However, the size of the grains and lattice strain of alloyed powder were determined by the Williamson–Hall method [33]. The size and morphology of powder particles were changed with milling time. Figure 3 presents the variation in the grain size of powder particles during MA and morphology of powder particles after MA of 12 h. Figure 3a shows how the grain size and lattice strain varied with the milling time. As the milling time increased, the powder particle size was reduced. The particle size decreased to 250 nm and the lattice strain was about 0.22%, after milling for 4 h. On the other hand, after milling of 12 h, the powder size was reduced notably to 75 nm and the lattice strain was 0.14%. The lattice strain increased, as the milling time increased. The increase in lattice strain is attributed to the increase in the lattice imperfections, such as grain boundaries and dislocation density. The morphology of the mixture observed by FE-SEM (JEOL 7600F, Tokyo, Japan) showed that no diffusion occurred at higher localized temperatures over the processing span. Figure 3b–d show the morphology of alloyed powder after 12 h of milling and it can be clearly seen that the powder size was significantly reduced to <75 nm.

### 3.2. Microstructure

**Figure 4:**
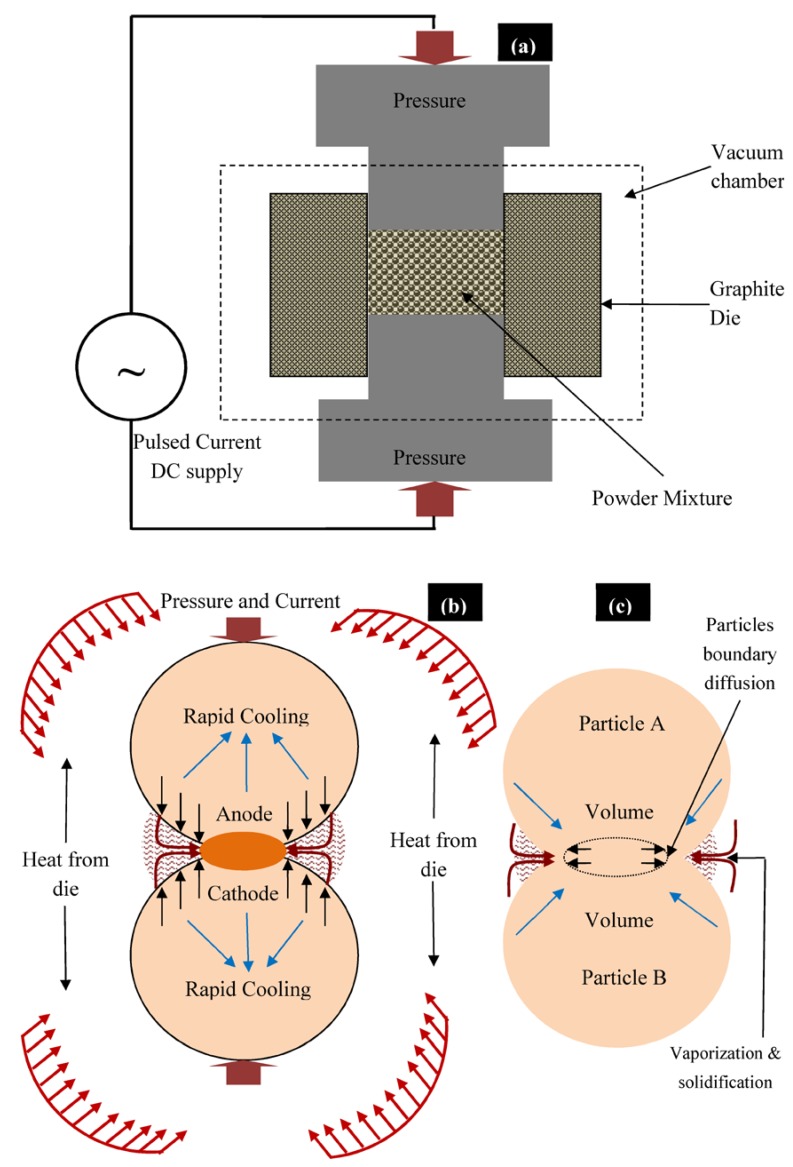
(**a**) Schematic representation of SPS technique and (**b**,**c**) mechanism of sintering of powder particles during the SPS process.

**Figure 5:**
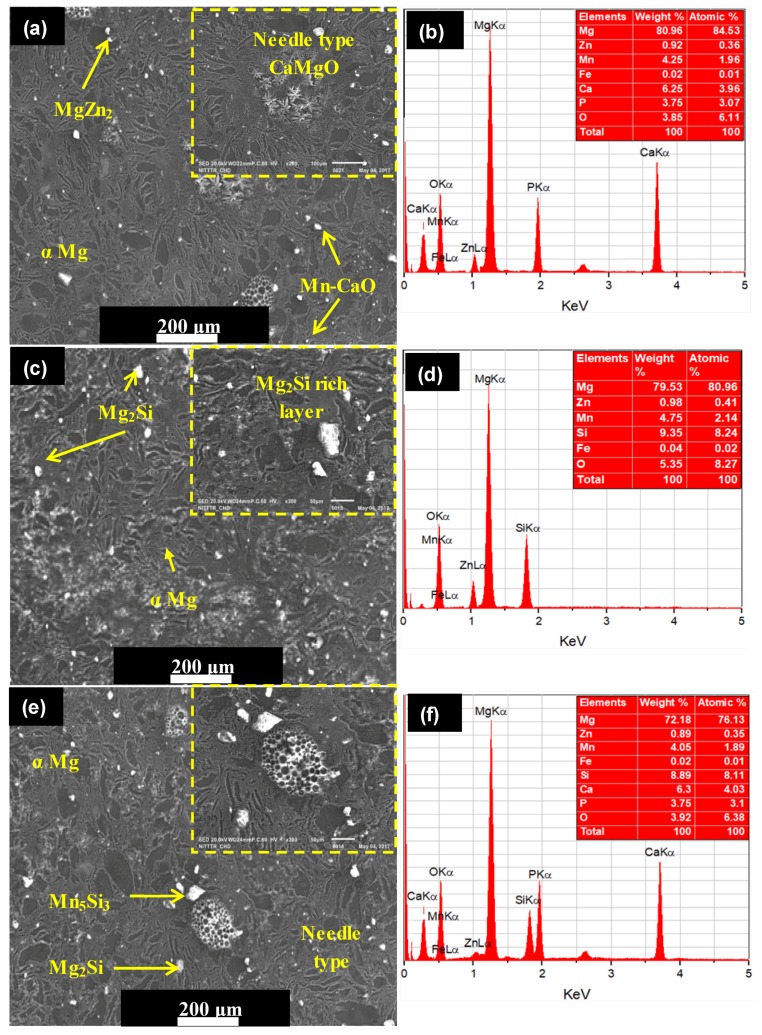
SEM micrographs and associated EDS spectra of as-fabricated composites sintered at a pressure of 50 MPa and a sintering temperature of 400 °C: (**a**,**b**) Mg–Zn–Mn–Si; (**c**,**d**) Mg–Zn–Mn–HA; and (**e**,**f**) Mg–Zn–Mn–Si–HA.

The structural morphology of the synthesized composites was directly dependent on the sintering temperature and applied pressure. As the sintering temperature and pressure increased, the densification of sintered green compact was increased. The high value of sintering pressure induces the high driving force, which helps in densifying or compacting the powder particles. Reportedly elevated sintering temperature assists the coalescence of the powder and reduces the porosity [30,31]. Figure 4a presents the sintering of powder particles during the SPS process. During the SPS process, thermal energy was generated due to electrical sparks between the powder particles and the contact area caused partial melting of the grain boundary of powder while uniaxially applied pressure densified the powder mixture (Figure 4b). The process of densification and solidification formed the final sintered compact. Figure 4c shows the mass transformation during the SPS process and the phenomena of partial diffusion and welding of powder particles as presented by Zheng et al. [32]. Three types of Mg-based composites, Mg–Zn–Mn–HA (Type-I), Mg–Zn–Mn–Si (Type-II), and Mg–Zn–Mn–Si–HA(Type-III) were synthesized. Figure 5 presents the microstructures and EDS spectra of all type of composites at a sintering pressure and a temperature of 40 MPa and 400 °C, respectively. Evidently, all three composites were completely densified and exhibited a low degree of structural porosity. With the change in element alloying composition (Si and HA), a distinct morphology can be observed in the composites. A thin and sharp needle-like laminar structure was observed as-distributed along the grain boundaries in Type-I composite (Figure 5a). Sunil et al. reported similar observations on HA addition in Mg composite, which enhances corrosion resistance [25]. This is attributed to the fact that the individual Mg flakes bonded together with HA and formed the layer-by-layer laminar structure in the form of needle (MgCaO).

EDS analysis indicated the element composition of Type-I composite and Fe, Ca, P, and O elements appeared with other elements (Mg, Zn, and Mn), as can be observed in Figure 5b. The peak intensities of Ca, P, and O elements confirmed the uniform distribution of HA in the composite. The SPS does not allow the oxidation during the sintering process. This is because the finer powder particle reacts quickly at room temperature. Therefore, the possible reaction behind the appearance of O element in the as-fabricated alloys resulted from handling of powder sample after milling and before sintering. The uniformly distributed HA in the composite leads to increase in the corrosion resistance [29,30,31]. On the other hand, when Si was used as an alloying element instead of HA, the typical change in the structure has been witnessed. When Si was used instead of HA, a mixture of discontinuous laminar and eutectic structure was observed. The microstructure of Type-II composite mainly comprised α–Mg, MgZn_2_, MnSi, and Mg_2_Si stages, as shown in Figure 5c. The Zn and MgZn_2_ existed as a hexagonally packed structure and a secondary phase, respectively. The MgZn_2_ phases were observed as an agglomeration of the compact fleck. The intermetallics Mn_5_Si_3_ and Mg_2_Si phases appeared in polygonal shape and can be clearly identified at the high magnification (×300). Ben-Hamu et al. observed the similar microstructure [24]. The associated EDS spectra confirmed the appearance of Si with other elements (Mg, Zn, Mn, Fe, and O), as illustrated in Figure 5d. When Si and HA were added in the Mg–Zn–Mn composite, the microstructure showed different morphologies (refer to Figure 5e). When HA and Si were used jointly as alloying elements, the appearance of needle-like structure can be clearly seen. Dark, gray and bright phases were identified as Mg matrix, CaMgSi, and Mg_2_Si phase. The typical eutectic structure disappeared and needle-like MgCaO phases formed. EDS analysis indicates the element composition of Type-III composite and Fe, Si, Ca, P, and O elements appeared with other elements (Mg, Zn, and Mn), as can be observed in Figure 5f.

**Figure 6:**
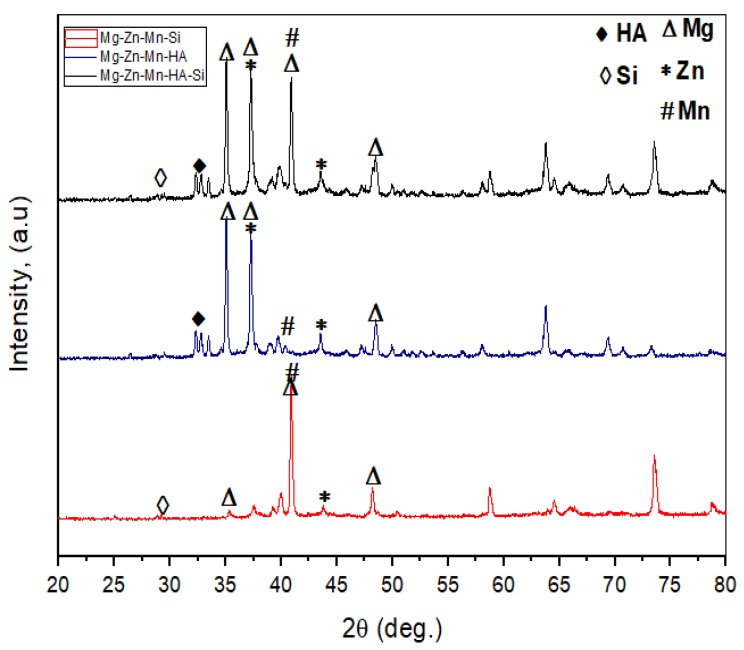
X-ray diffraction patterns of all types of as-fabricated Mg composites.

The XRD patterns of all types of sintered composites are presented in Figure 6. It can be observed that all sintered composites had the same XRD pattern; however, their respective peak intensities changed with the weight percentages of Si and HA. Biocompatible and biomimitic phases were identified in the sintered composites. MgCaO, Mn–CaO, and CaMgZn phases were observed in the Type-I composite. The Type-II composite comprised Mg_2_Si, Mg0.97Zn0.03, and Mn_5_Si_3_ phases. Mg_2_Si was expected to enhance the corrosion resistance. The Type-III composite showed CaMgSi, Mg_2_Si, Mn_5_Si_3_, Mn–CaO, and CaMgZn phases, which are beneficial to form the apatite growth and improve the bioactivity.

### 3.3. Mechanical Properties

**Figure 7:**
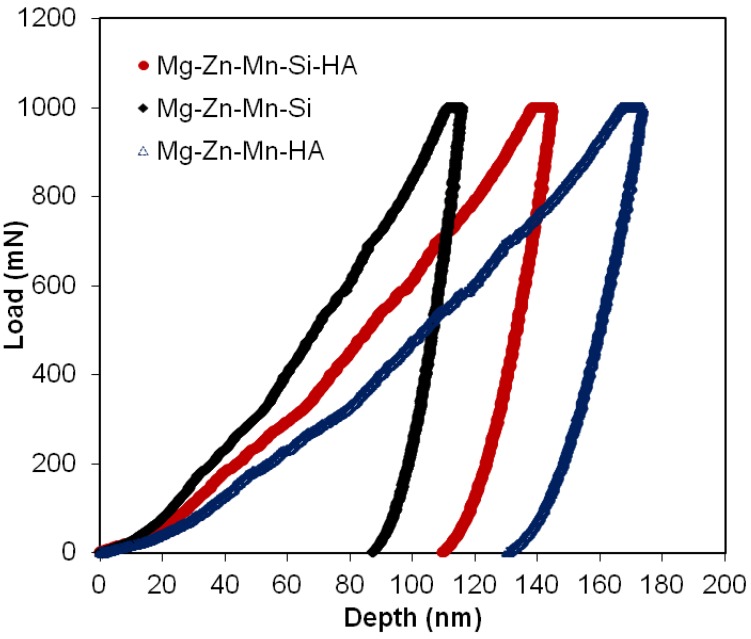
Load–depth curves of as-fabricated all types of Mg composites.

Figure 7 shows the distinctive loading/unloading plots for all types of sintered composites. Table 3 presents mechanical properties of all three composite composites. The Type-I composite (Mg–Zn–Mn–HA) showed low elastic modulus and hardness, which were estimated to be 32 GPa and 0.54 GPA, respectively. The Type-I composite exhibited low hardness due to high degree of structural porosity. The high degree of porosity in structure causes the reduction in mechanical properties of the bulk material. When Si was used as an alloying element instead of HA, the densification of bulk increased and the mechanical properties of compact were improved in terms of hardness and elastic modulus. The Type-II composite (Mg–Zn–Mn–Si) offered high values of elastic modulus and hardness, which were estimated to be about 45 GPa and 1.97 GPa, respectively. When Si and HA were used jointly as alloying elements, the degree level of porosity increased again, which led to the reduction in hardness and elastic modulus again. The hardness of as-synthesized alloys was higher than the pure Mg. The increase in the hardness of bulk material is due to cold hardening of Mg as well as due to the presence of HA and MgO at inter-laminar sites. Notably, the elastic modulus and hardness for the Type-III composite were 39 GPa and 1.18 GPa, which were smaller than the Type-II composite but higher than the Type-I composite, as seen in Table 3.

### 3.4. In Vitro Corrosion and Degradation Analysis

**Figure 8:**
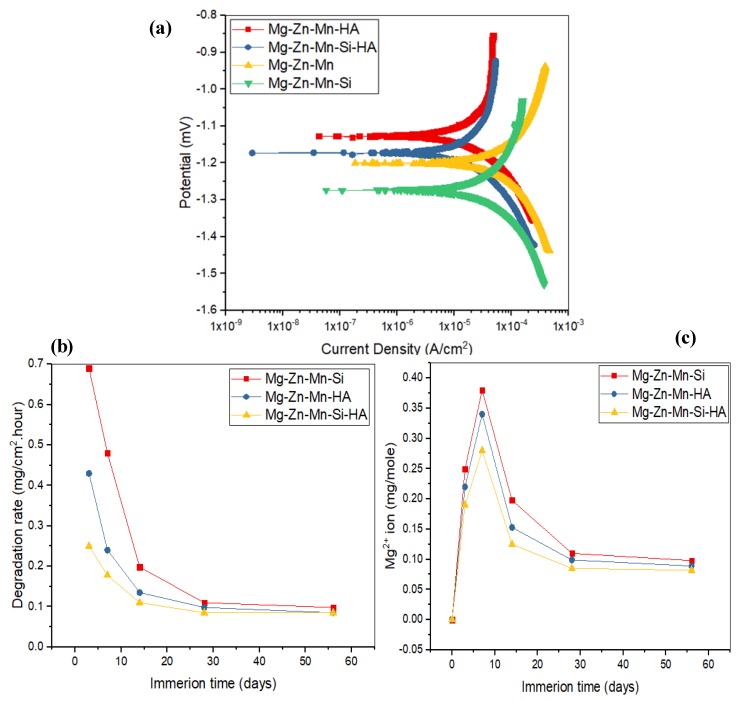
(**a**) Potential dynamic polarization curves of Mg–Zn–Mn, Mg–Zn–Mn–Si, Mg–Zn–Mn–HA, and Mg–Zn–Mn–Si–HA alloys at (37 ± 1) °C; (**b**) degradation rate of composites as a function of time, and (**c**) concentrations of Mg^2+^ of composites in the simulated body fluid (SBF) medium.

In vitro corrosion characteristics and degradation behavior of the as-fabricated composites were assessed by a Tafel extrapolation method and an immersion test. Figure 8 presents the corrosion characteristics and degradation behavior of as-fabricated composites. Figure 8a illustrates a comparison of corrosion Tafel polarization curves of all synthesized composites. Table 4 presents the determined corrosion characteristics, such as the corrosion potential (E_corr_), corrosion current density (I_corr_), polarization resistance (R_p_), and corrosion rate (C_R_) for all types of materials. From the investigation, it can be seen that cathodic and anodic reactions obtained were same for all types of specimens, which are the typical characteristics of passive behavior. The corrosion parameters for Mg–Zn–Mn, such as I_corr_ and E_corr_, were measured to be 19.5 μA/cm^2^ and −1.2 mV, respectively. The corrosion current density was very low as compared to all specimens; the samples had least corrosion resistance and the increased degradation was 1.98 mm/year. When the Si was alloyed in Mg–Zn–Mn, the corresponding current density and corrosion potential were measured around 7.7 μA/cm^2^ and −1.27 mV, respectively. The Type-II composite possessed higher corrosion resistance as compared to Mg–Zn–Mn and the corrosion rate was measured to be around 1.45 mm/year. However, the Type-II alloy still had low corrosion resistance and the alloying of Si element was less preventive from corrosion. When HA was used as an alloying element, the hyperbolic curve was shifted slightly towards the lower current density, and the corresponding current density and corrosion potential were measured to be around 3.5 μA/cm^2^ and −1.13 mV, respectively. The Type-I composite possessed better corrosion resistance as compared to Mg–Zn–Mn and the Type-II composite. The corresponding corrosion rate was measured to be around 0.97 mm/year. The alloying of HA element in Mg–Zn–Mn increased the corrosion resistance. This is attributed to the formation of corrosion barrier phases (CaMg and Mg0.97Zn0.03) in the composite that promoted the apatite layer growth on the composite surface, which resisted the degradation/corrosion of composite in the SBF medium. The corrosion morphology of Mg–Zn–Mn–HA composite samples was found less corroded as compared to the Mg–Zn–Mn–Si composite (Figure 8b). On the other hand, when both HA and Si were used as alloying elements, excellent corrosion resistance was offered by the specimen, and the corresponding current density and corrosion potential were measured to be around 0.98 μA/cm^2^ and −1.17 mV, respectively. The Mg–Zn–Mn–Si–HA composite possessed better corrosion resistance as compared to all other types of as-sintered composites, and the corrosion rate was measured to be around 0.15 mm/year. The above observed finding suggested that the Type-III composite can hold up the degradation rate at a pace that matches the period of bone healing, which is the prime objective of the current study.

Figure 8b represents the degradation behavior of the as-fabricated Mg composite specimens in SBF. It has been found that during the initial period, the degradation rate of all-sintered alloys was high, but no further effects were seen after 28 days. Comparatively, the degradation rate of Type-II alloy was high as compared to the Type-I and Type-III composites. When 10% HA and Si were used as a reinforcement, the rate of mass deposition of apatite layer was high as compared to the Type-I and Type-II composite samples. Figure 8c illustrates the Mg^2+^ concentration in the SBF solution. In the early phase of immersion test (up to 7 days), the release of Mg^2+^ was higher, but after 7 days, the release rate of Mg^2+^ began reducing as a result of deposition of a thick apatite layer on the surface of specimens. The Mg^2+^ dissolution was found larger for Mg–Zn–Mn–Si specimens among all types of composites, presenting high degradation, which showed a similar trend as found in the degradation rate (Figure 8b). Furthermore, when Si and HA elements were used as alloying elements, a very significant and drastic reduction in the dissolution of Mg^2+^ ion was found, as can be seen from Figure 8c.

**Figure 9:**
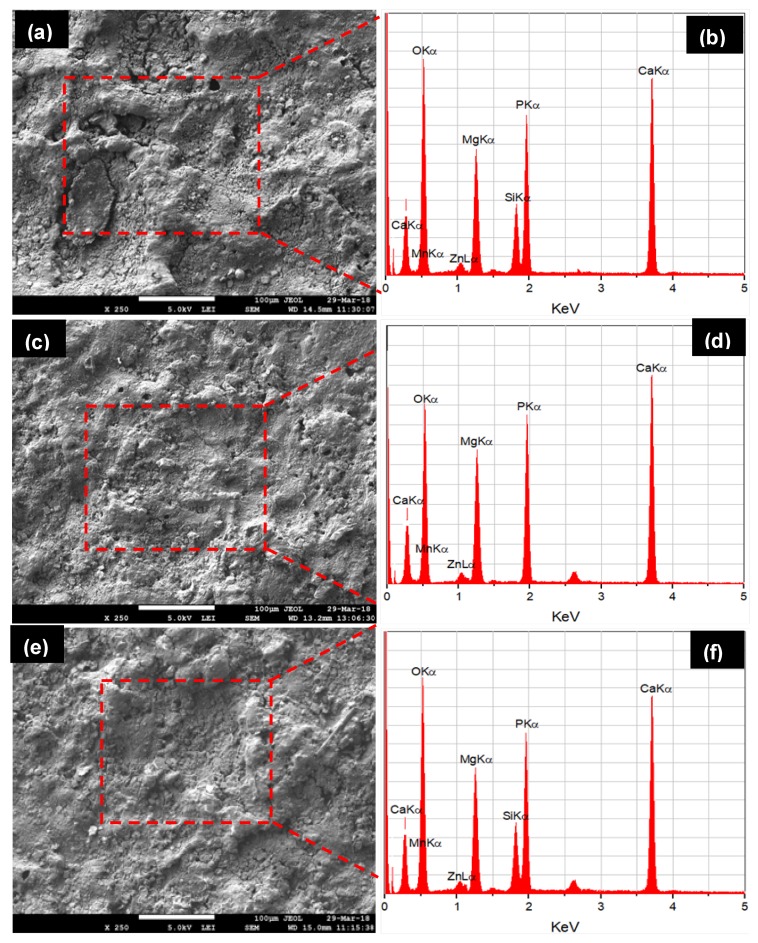
SEM micrographs and EDS spectra of the degraded morphology of composites after 28 days in the SBF immersion.

Figure 9 shows the corroded morphologies and EDS spectra of the all types of samples after 28 days of immersion in the SBF solution. The Type-II alloy surface was found to be highly corroded. This is because the developed apatite layer on the composite surface was weaker and therefore degraded rapidly in the SBF medium. The apatite layer was shredded due to its highly porous nature and degradation took place in the form of pulverized fine particles, as can be seen in Figure 9a. Open holes, cracks, and shredded layers were clearly seen on the corroded surface due to release of H_2_ gas and Mg^2+^ ions. The shredding of apatite and traces of pulverized Ca and P particles can be easily identified as holes/cracks. The growth of apatite layer formation was confirmed by EDS-analysis, as can be seen in Figure 9b. When HA was used as an alloying element, the composite sample was less corroded as compared to the Mg–Zn–Mn–Si composite. The apatite layer growth on the composite (Type-I) surface was high as compared to the Type-II composite, which resisted the degradation/corrosion of composite in the SBF medium. This is attributed to the formation of corrosion barrier phases (CaMg and Mg0.97 Zn0.03) in the composite. The corrosion morphology of Mg–Zn–Mn–HA composite sample was seen in Figure 9c. Still, open holes, shredding of apatite layer and traces of pulverised materials were found and high peaks of Ca and P in the associated EDS spectrum confirmed the formation of the thick layer of apatite growth, as can be seen in Figure 9d. Samples with hybrid and proportionate filling of HA and Si elements showed better corrosion resistance. The Mg–Zn–Mn–Si–HA composite had the least corrosion rate as compared to Mg–Zn–Mn–Si and Mg–Zn–Mn–HA composites, as can be seen in Figure 9e. This is because a very thick apatite layer was developed on the composites’ surface, which resisted it from degradation and the presence of CaMgSi, Mg_2_Si, Mn_5_Si_3_, Mn–CaO, CaMgZn, and MnSi phases fortified the mechano-corrosion and biological properties. Figure 9f presents the corroded surface morphology of the Mg–Zn–Mn–Si–HA composite and a pulverized surface with comparatively less holes was observed. 

### 3.5. In Vitro Biocompatibility Assessment

**Figure 10:**
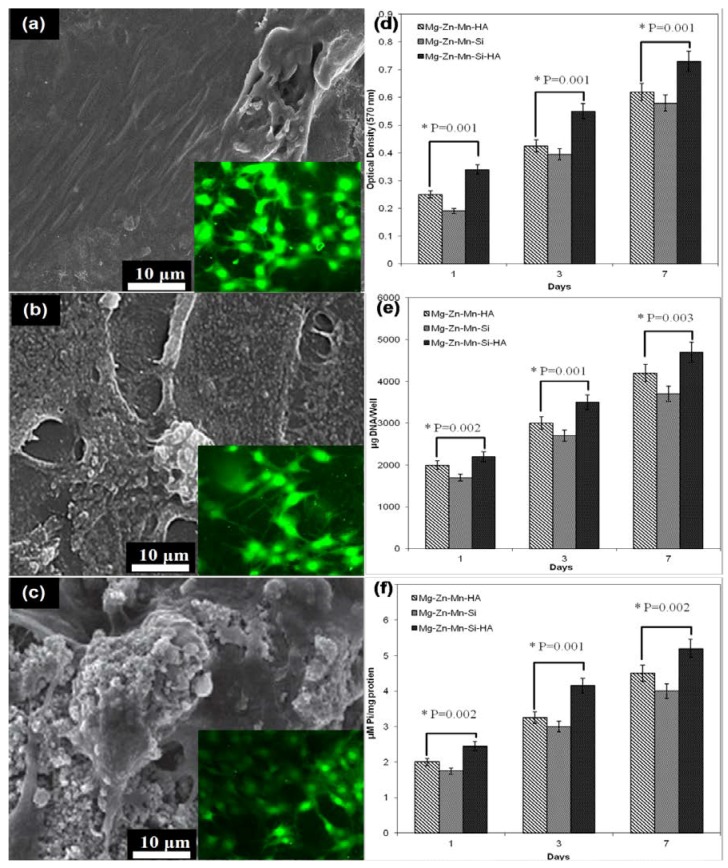
MG-63 cell adhesion after 24 h: (**a**) Mg–Zn–Si; (**b**) Mg–Zn–Mn–HA and (**c**) Mg–Zn–Mn–Si–HA surface and cell proliferation and differentiation: (**d**) MTT assay; (**e**) DNA content and (**f**) alkaline phosphatase-specific (ALP) activity of MG-63 cells determined on Days 1, 3, and 7 (individual group was statistically highly significant (*p* < 0.001)).

The structural morphology and elemental composition of implant played a very important role in establishing the bio-mechanical bonding between the implant surface and surrounding tissues. A number of studies reported that HA has significant influence on the adhesion and growth of cells [29,35,38]. Recently, the alloying of Si and HA elements is found favourable for the enhancement of bioactivity of Mg alloys and composites [29,30,31]. Figure 10 presents the fluorescent fluorescence staining, cell attachment, proliferation activities and differentiation activities of osetoblatic cell (MG-63) on the as-synthesized Mg–Zn–Mn–(HA–Si) composites. With the increase in incubation time, the adhesion and proliferation of MG-63 cells increased significantly.

Figure 10a–c present the attached cell morphology and fluorescent staining on the Type-I, Type-II, and type-III composites, respectively. Fluorescent staining is generally used to indicate intracellular esterase activity present in viable cells. Dense and evenly dispersed multi-layered cells with large nuclei were observed for all samples; however, in the case of Mg–Zn–Mn–(HA–Si) samples, there were larger numbers of living cells. Compositionally, the reinforcement of HA had very significant impact on the apatite-inducing ability and bioactivity of implant. Moreover, spontaneous formation of bio-compatible phases of composition CaMgSi, Mg_2_Si, Mn_5_Si_3_, Mn–CaO, CaMgZn, and MnSi, provided a biomimetic inert layer on the alloy surface, which accelerated the bone adhesion, proliferation, growth and differentiation of MG-63 cell line. Moreover, porous structure leads to the formation of hydrophilic surface and provides a vehicle and mechanical anchoring sites to interact with cells. In the current study, the as-sintered porous Mg–Zn–Mn–(Si–HA) alloys possessed micro-scale pore structures ranging from 20–50 μm mimicking human bone, which met the requirement of osseiointergation. After coming in the contact with the composite surface, the MG-63 cells started adhering on the surface, and after 24 h, cells started spreading. The shape of cells mainly elongated and polygonal which indicated that cells were well adhered, spread and proliferated. Polygonal-shape cells represented the excellent adhesion and growth on the as-synthesized composites surface. A number of activities, such as filopodias, lamellipodia, and peripheral ruffles, were seen. Figure 9d–f present the cell proliferation, DNA content, and alkaline phosphatase-specific (ALP) differentiation activities. All observed data was statically analyzed at a 95% confidence level using ANOVA, and individual group was statistically highly significant (*p* < 0.001) for each treatment (different alloy compositions) at different time intervals (days). Higher numbers of cells were grown on the Type-III composite surface. The optical density showed the proliferation of MG-63 cells on the composite test specimens, as presented in Figure 9d. The Type-III composite surface possessed a higher cell proliferation rate. This is attributed to the presence of Si and HA elements, which enhanced the bone formation process. Moreover, the structural porosities escalated the surface energy, which promoted protein absorption and cell growth. The DNA content on the specimen’s surface increased with the increase in the proliferation rate, as can be seen that the Type-III composite specimens had a higher proportion of DNA content (Figure 9e). The ALP-type differentiation activities of MG-63 cells were presented in Figure 9f. The serum level of ALP activity was found significantly higher in the Type-III composite specimens, compared with the Type-II and Type-I composite specimens.

## 4. Conclusions

Biomimetic, biodegradable, low elastic and mechanically tuned Mg–Zn–Mn–(Si–HA) composites were fabricated by the element alloying and SPS technique. The investigation revealed that pore characteristics of size ranging from 25–50 μm, and 20–30% porosity has been achieved by adding HA and Si from 5 wt %. The Mg–Zn–Mn–(Si–HA) alloys possessed not only porous structure, but also possessed low elastic modulus ranging from 15 to 30 GPa that helped in reducing the stress shielding effect. Further, the developed alloys attained reasonable hardness ranging from 86–200 HV. The alloying of HA and Si elements led to the formation of biomimetic and biocompatible phases, such as CaMg, MgSi2, Mg–Zn, Mn–Si, Mn–CaO, Mn–P, Ca–Mn–O, and CaMgSi in the porous layers, which enhanced the corrosion characteristics of the alloys. Moreover, the appearance of Ca, P and O elements in the EDS spectrum conferred the bioactivity of the as-synthesized alloys. The in vitro bioactivity results indicated that the Mg–Zn–Mn–HA–Si alloy had excellent biocompatibility and promoted cell adhesion, growth, proliferation, and differentiation.

## Figures and Tables

**Table 1:** Composition of alloying elements in wt % as-proposed for bio-composites.

Composite	Composition	Alloying Element Composition, wt %				
		Zn	Mn	Si	HA	Mg
Type-I	Mg–Zn–Mn–HA	1	5		10	Bal.
Type-II	Mg–Zn–Mn–Si	1	5	10		Bal.
Type-III	Mg–Zn–Mn–HA–Si	1	5	10	10	Bal.

**Table 2:** Process parameters of the mechanical alloying assisted SPS and their levels.

Process Parameters	Symbol	Units	Levels
Type of alloying element	A_e_		HA, Si, Si–HA
Milling time, h	T_m_	h	4, 8, 12
Sintering pressure	P_s_	MPa	30, 40, 50
Sintering temperature	T_s_	°C	350, 400, 450
Heating rate		°C/min	50
Holding time		Min	5
Atmosphere			Argon

**Table 3:** Elastic modulus and hardness of the sintered biocomposites.

Mg Alloys	Mechanical Properties			
	Elastic Modulus, E (GPa)		Hardness, H (GPa)	
	Mean of Sample Group	Standard Deviation	Mean of Sample Group	Standard Deviation
Mg–Zn–Mn–HA	32	1.58	0.54	0.02
Mg–Zn–Mn–Si	45	2.64	1.97	0.03
Mg–Zn–Mn–Si–HA	39	1.98	1.18	0.02

**Table 4:** Corrosion parameters determined by the Tafel extrapolation method.

Parameters	Mg Alloys			
	Mg–Zn–Mn	Mg–Zn–Mn–Si	Mg–Zn–Mn–HA	Mg–Zn–Mn–Si–HA
*I*_corr_ (µA/cm^2^)	22.7	7.7	3.3	0.98
*E*_corr_ (mV )	−1.27	−1.27	−1.13	−1.17
*C*_R_ (mm/year)	1.98	1.45	0.97	0.15
